# Supplementary material for: Classroom Movement Breaks and Physically Active Learning Are Feasible, Reduce Sedentary Behaviour and Fatigue, and May Increase Focus in University Students: A Systematic Review and Meta-Analysis
Source: Int J Environ Res Public Health. 2022 Jun 24;19(13):7775. doi: 10.3390/ijerph19137775 (PMC9265656; doi:10.3390/ijerph19137775)
Supplement: Supplementary file 1 [file ijerph-19-07775-s001.zip › ijerph-1770939-supplementary.pdf]

**Medline**

CONCEPT 1 Intervention

movement break* OR exercis* Break* OR active learning OR active lesson* OR education outside of the classroom OR Acute exercise OR Acute Physical activity OR High-intensity interval activity OR High-intensity interval training OR High Intensity Interval Exercise OR Mov* and academic learning OR brain break* OR activ* break* OR Physical* active learning OR activ* workstation* OR sit-to-stand desk* OR sit-stand desk* OR breaks in sitting time OR classroom intervention OR stationary cycl* OR walk* break OR breaking up prolonged sitting OR height-adjustable desk* OR sedentary break*

AND

CONCEPT 2 Population

Tertiary OR college OR universit* OR higher education OR higher learning OR lecture* OR tutorial* OR workshop* OR lecture-based OR tutorial-based OR workshop-based

**CINAHL**

CONCEPT 1 Intervention

movement break* OR exercis* Break* OR active learning OR active lesson* OR education outside of the classroom OR Acute exercise OR Acute Physical activity OR High-intensity interval activity OR High-intensity interval training OR High Intensity Interval Exercise OR Mov* and academic learning OR brain break* OR activ* break* OR Physical* active learning OR activ* workstation* OR sit-to-stand desk* OR sit-stand desk* OR breaks in sitting time OR classroom intervention OR stationary cycl* OR walk* break OR breaking up prolonged sitting OR height-adjustable desk* OR sedentary break*

AND

CONCEPT 2 Population

Tertiary OR college OR universit* OR higher education OR higher learning OR lecture* OR tutorial* OR workshop* OR lecture-based OR tutorial-based OR workshop-based

**Embase**

CONCEPT 1 Intervention

movement break* OR exercis* Break* OR active learning OR active lesson* OR education outside of the classroom OR Acute exercise OR Acute Physical activity OR High-intensity interval activity OR High-intensity interval training OR High Intensity Interval Exercise OR Mov* and academic learning OR brain break* OR activ* break* OR Physical* active learning OR activ* workstation* OR sit-to-stand desk* OR sit-stand desk* OR breaks in sitting time OR classroom intervention OR stationary cycl* OR walk* break OR breaking up prolonged sitting OR height-adjustable desk* OR sedentary break*

AND

CONCEPT 2 Population

Tertiary OR college OR universit* OR higher education OR higher learning OR lecture* OR tutorial* OR workshop* OR lecture-based OR tutorial-based OR workshop-based

**Psychoinfo**

CONCEPT 1 Intervention

movement break* OR exercis* Break* OR active learning OR active lesson* OR education outside of the classroom OR Acute exercise OR Acute Physical activity OR High-intensity interval activity OR High-intensity interval training OR High Intensity Interval Exercise OR Mov* and academic learning OR brain break* OR activ* break* OR Physical* active learning OR activ* workstation* OR sit-to-stand desk* OR sit-stand desk* OR breaks in sitting time OR classroom intervention OR stationary cycl* OR walk* break OR breaking up prolonged sitting OR height-adjustable desk* OR sedentary break*

AND

CONCEPT 2 Population

Tertiary OR college OR universit* OR higher education OR higher learning OR lecture* OR tutorial* OR workshop* OR lecture-based OR tutorial-based OR workshop-based

**PUBMED**

CONCEPT 1 Intervention

"movement break*" OR "exercise break*" OR "brain break*" OR "active break*" OR "Physically active learning"

AND

CONCEPT 2 Population

Tertiary OR college OR universit*
